# Analysis of structural variations and effects of the national volume-based procurement policy on inpatient costs: a real world study of osteoporotic hip fractures in Shanghai, China

**DOI:** 10.3389/fpubh.2025.1678817

**Published:** 2025-10-30

**Authors:** Jing Yan, Qiujun Qin, Xinye Fang, Fen Li, Bifan Zhu

**Affiliations:** ^1^Shanghai Health Development Research Center (Shanghai Medical Information Center), Shanghai, China; ^2^School of Public Health, Fudan University, Shanghai, China; ^3^Financial Management Affairs Center of Shanghai Municipal Health Commission, Shanghai, China

**Keywords:** osteoporotic hip fractures, medical cost, structural variation, osteoporosis, price policy

## Abstract

**Background:**

As one of the most severe clinical manifestations of osteoporosis, osteoporotic hip fractures often require surgical treatment and result in high medical costs. The hospitalization expenses for patients with osteoporotic hip fractures in China have been on the rise; however, there are few reports on the structural variations in hospitalization costs for these patients and the correlation between total and individual expenses. This research aims to analyze the structure of hospitalization costs and their changes over an extended period in Shanghai, China, and to investigate the impact of the national volume-based procurement (NVBP) policy on costs associated with osteoporotic hip fractures.

**Methods:**

Data were obtained from the Shanghai Health Statistics Center, which includes hospitalization records for patients at all medical institutions in Shanghai from January 2017 to December 2022. Structural variation analysis was utilized to assess the shifts in yearly hospitalization cost composition, while new gray correlation analysis was employed to explore the degree of association between individual and total costs across both overall and various levels of hospitals. Additionally, we evaluated the effects of the NVBP on total and individual hospitalization costs using interrupted time series analysis.

**Results:**

While there was a decline in 2022, medical consumable costs accounted for over 59% of total inpatient costs for osteoporotic hip fractures and consistently demonstrated the highest correlation with total hospitalization costs in Shanghai from 2017 to 2021. Drug costs not only had the highest contribution rate to overall inpatient expenses during the years 2017–2018 (44.22%) and 2020–2021 (36.76%) but also held the top position in the contribution rate of structural variation (CRSV) from 2017 to 2022 (45.77%). The stratification results at the hospital level indicated that drug costs in tertiary and secondary hospitals declined over 6 years, while primary hospitals only saw reductions in a few years. Furthermore, medical consumable costs for tertiary and secondary hospitals decreased in 2021–2022, with CRSVs of 23.36 and 37.91%; however, primary hospitals had higher medical consumable costs during this period. After the NVBP policy was implemented, total hospitalization costs significantly decreased by 5,022.088 yuan (*p* < 0.001). Additionally, hospitalization costs exhibited a significant downward trend over time, decreasing by 596.114 yuan after the intervention (*p* < 0.001). Medical consumable costs also exhibited a declining pattern regarding both immediate effects and long-term patterns after the reform, with an average decrease of 290.448 yuan (*p* < 0.001).

**Conclusion:**

Although the medical consumable costs related to osteoporotic hip fractures made up the largest portion of total expenditures for inpatients, their share declined due to the implementation of the NVBP. Despite the significant structural variation in drug costs, they still represented the second-largest part of total hospitalization expenditures. Future policy should focus on addressing the rising hospitalization costs of osteoporotic hip fractures, particularly those associated with medical consumables and drugs. Besides, additional regulations should be established based on the cost structures of medical institutions at various levels.

## 1 Introduction

Osteoporosis is a systemic bone disease marked by low bone mass, deterioration of the bone microstructure, increased bone fragility, and increased risk of fractures (1, 2). In 2019, osteoporosis was predicted to affect 25.5 million women and 6.5 million men in the European Union, including Switzerland and the United Kingdom (3). An epidemiological study conducted in China shows that the overall osteoporosis prevalence in women aged 40 and older is 20.6%, compared to 5.0% in men within the same age group (4). Additionally, postmenopausal women and men aged 50 and older exhibit higher osteoporosis rates, at 32.1% and 6.9%, respectively (4). Osteoporotic fracture is a critical sign of osteoporosis. Half of women and one in five men might suffer from a fragility fracture in their lifetime beyond the age of 50, making osteoporotic fractures a serious public health concern (4). Hip, forearm, humeral, and vertebral fractures are the main osteoporotic fracture sites (5–7). Hip fractures are regarded as a nation’s indicator of osteoporosis and a helpful proxy for calculating the global osteoporosis burden (8). The primary cause of hip fractures is falls, which is associated with low bone density (9, 10). Hip fractures are among the most serious osteoporotic fractures; they are painful and almost always require surgery and hospitalization (11, 12). In the first year following a hip fracture, up to 20% of patients die, mainly due to severe underlying medical issues, and fewer than half of the survivors return to their pre-hip fracture level of function (12).

Osteoporotic hip fractures have the highest hospitalization costs compared to vertebral and wrist fractures, imposing considerable clinical and financial strains on the healthcare system (13). A study from Canada found that, 5 years after a hip fracture, healthcare costs for patients remained higher than before the fracture, with median costs of $12,670 for women and $7,933 for men (14). Between 2014 and 2020, the overall cost of hospitalization in Ireland for hip fractures was approximately €296 million, with the annual cost increasing from around €34 million in 2014 to €44 million in 2020 (15). Hospitalization costs for hip fracture patients in Shanghai increased from 2015 to 2020, with over 90% opting for surgery (16). In China, medical consumables costs make up the largest portion of hospitalization costs for osteoporotic fracture patients, with medicine costs second (17–19). High medical costs can exacerbate healthcare inequalities, influenced by social factors such as income and education levels (20, 21). To tackle the increasing healthcare costs, many countries have implemented price and purchasing policies that mainly focus on establishing reference pricing systems, regulating doctors’ prescribing behaviors, and volume-based pricing and procurement policies (22–24).

In China, several policies have been implemented to manage medical expenses for osteoporotic hip fractures, including the zero-markup drug policy, adjustments to medical services prices, and restrictions on medical consumable markup rates, zero-markup policy for medical consumables, and national volume-based procurement (NVBP) policy (19, 25–33). In 2019, a policy was implemented in Shanghai, China, mandating that public medical institutions sell medical consumables at the current purchase price, without any increase (31). Furthermore, guidance on the development of NVBP and the use of high-value medical consumables was created in 2021 by the Chinese government, and the NVBP for artificial joints was initiated in Shanghai on June 30, 2022 (25, 31). In addition to controlling medical consumable costs, the Chinese government has also introduced a series of cost-control policies regarding drug expenses. The fixed percentage markup drug policy was introduced in Shanghai, China, to eliminate a 10–15% markup on drugs as a transitional measure (19, 26, 27, 29). Following this, the zero mark-up policy on all drugs was fully implemented in 2017, which means drug mark-ups are no longer used to compensate healthcare providers, thereby reducing irrational expenditures.

Previous studies have only reported the overall trend of hospitalization costs for osteoporotic hip fracture patients, but lacked an analysis of the structure of these costs and their variations over different years, which may mask unreasonable increases in certain individual expenses and make it difficult to provide a basis for policy formulation and evaluation (11, 16). Meanwhile, most studies calculated only the proportion of each cost category without examining the degree of association between individual cost categories and total hospitalization costs, making it challenging to reflect the dynamic relationship between these expenses (18, 34). Additionally, earlier research has primarily focused on the medical expenses at tertiary hospitals, leaving a gap in reporting on the healthcare costs of inpatients in primary and secondary hospitals and the changes in their cost structures over an extended period (13, 35, 36). Furthermore, current studies have emphasized analyzing the impact of drug price policies, whereas only a few have utilized interrupted time series to explore the NVBP for medical consumables (19, 27, 29). Therefore, this study analyzed the structure and long-term trends of hospitalization costs for osteoporotic hip fracture inpatients from 2017 to 2022. We used gray correlation analysis to identify key cost components and categorized hospitals by level to examine shifts in cost composition and correlations among hospital types. The study also discussed policy factors influencing these changes and assessed the impact of the NVBP on costs.

## 2 Methods

### 2.1 Data source

The study data were obtained from the Shanghai Health Statistics Center, which includes hospitalization records for patients at all medical institutions in Shanghai from 2017 to 2022. The data in this study included gender, age, length of admission, disease code, level of surgery, name of surgery and operation, type of insurance, and cost of hospitalization. Patients with osteoporotic hip fractures were identified based on ICD-10 codes.

### 2.2 Study population

The inclusion criteria of the study sample were as follows: admission during the follow-up period (2017–2022) with hip fracture diagnostic codes (ICD-10 code: S72). The exclusion criteria were (1) pathologic diagnosis of high-energy injury (such as car accidents, falls from heights, etc.), (2) medical institution level 0, (3) missing information such as age, (4) the number of days between the discharge of the last hip fracture hospitalization and the next admission being less than 180 days, and (5) length of hospitalization less than 1 day. This study ultimately included 82,640 hospitalized patients with osteoporotic hip fractures.

### 2.3 Outcome measures

The total hospitalization costs included medical consumables (such as disposable medical fees for examinations, surgeries, and treatments), drugs (including antibiotics and other drugs), treatment (covering surgical and non-surgical procedures), diagnosis (encompassing pathology, laboratory, imaging, and clinical diagnosis), comprehensive medical services (consists of general medical services costs general treatment operation fees, nursing fees, and other charges), and other costs (containing rehabilitation, traditional Chinese medicine, blood and blood products fees, and additional charges). The hospitalization costs in this study were adjusted to 2022 constant prices based on the Consumer Price Index for Healthcare in Shanghai.

The classification of surgical level in this study is as follows: first-degree surgery is low-risk, simple operations with minimal technical difficulty; second-degree surgery carries some risk, possess average complexity, and present some technical challenges; third-degree surgery involves high risk, significant complexity, and substantial difficulty, requiring considerable resources; and fourth-degree surgery also has high risk and complexity, extreme difficulty, and often entail substantial ethical considerations (25).

### 2.4 Statistical analysis

#### 2.4.1 Structural variation analysis

The analysis of the degree of structural variations reflects trends in the internal structure of costs through three indicators: value of structure variation (VSV), degree of structure variation (DSV), and contribution rate of structural variation (CRSV) (37).

VSV is the primary indicator for evaluating the extent and direction of changes in the internal structure of total costs, representing the difference between the beginning and end of an item over a specified period. The formula is as follows:


$$\mathrm{VSV}=X_{i1}-X_{i0}$$

In the above formula, *i* represents the cost category, 0 indicates the beginning of a specific period, and 1 marks the end of that period. When VSV > 0, it indicates that the item’s proportion is increasing over time; otherwise, it suggests a decreasing trend.

DSV represents the total values of the changes in the cost structure of each category during a specific time, primarily reflecting the overall variations in cost category throughout that time frame. It is calculated in the following way:


DSV=∑∣VSV∣

A higher DSV indicates a larger degree of structural change over the period.

CRSV represents the absolute VSV of each cost category as a proportion of the degree of structural change. It measures the relative contribution of the VSV of each cost category to the overall degree of structural variation. The CRSV is calculated as:


$$\text{CRSV}=\frac{|X_{i1}-X_{i0}|}{\mathrm{DSV}}\times 100\%$$

The higher the CRSV, the more significant the cost category drives the total cost’s structural variation.

#### 2.4.2 New gray relational analysis

The new gray correlation analysis simplifies the steps in processing dimensionless data using the gray correlation analysis method. It directly calculates the correlation coefficient between each comparison sequence and the reference sequence by employing the absolute value difference, which more intuitively expresses the degree of correlation between the factors. The calculation steps are as follows:

- *Step 1*: Define the reference series and the comparison series.

Use the hospitalization costs as the reference series $X_{0}(k)$ , where *k* = 1–6, representing the years 2017–2022, respectively; use the individual cost category as the comparison series $X_{i}(k)$ , where *i* = 1–6, representing medical consumables, drugs, treatment, diagnosis, comprehensive medical services, and other costs, respectively.

- *Step 2*: Calculate the difference sequence between each comparison sequence and the reference sequence, and find the maximum and minimum absolute values of the differences.

$$\Delta_{i}(k)=|X_{i}(k)-X_{0}(k)|$$

- *Step 3*: Calculate the correlation coefficient.

$$R_{i}(k)=\frac{\Delta_{\min}(k)+\rho \Delta_{\max}(k)}{\Delta_{i}(k)+\rho \Delta_{\max}(k)},\text{where}\;\rho \;\text{is}\;\mathrm{set}\;\text{to}\;0.5$$

- *Step 4*: Calculate the correlation of individual cost category and rank them.

$$\gamma_{i}(k)=\frac{1}{n}\sum R_{i}(k),\text{where}\;n\;\text{is}\;\mathrm{set}\;\text{to}\;6$$

#### 2.4.3 Interrupted time series analysis

Interrupted time series design controls for the regression trend before the intervention to assess its impact on the series. It compares and tests the immediate level changes in the outcome variable before and after the intervention at the intervention point, as well as the changes in the regression slopes before and after the intervention, thereby evaluating the effectiveness of the intervention (38). This study used segmented linear regression model to regress the periods before and after the intervention. The model formula is as follows:


$$Y_{t}=\beta_{0}+\beta_{1}*X_{\text{time}}+\beta_{2}*X_{\text{intervention}}+\beta_{3}*X_{\text{posttime}}+\varepsilon_{t}$$

$Y_{t}$ is the average cost of hospitalization. $X_{\text{time}}$ is a time count variable. The unit of time in this study is half month, from January 2021 to December 2022, a total of 48 time points, with nodes “0, 1, 2……, 48”. $X_{\text{intervention}}$ refers to the dummy variable of whether the policy occurred, which is recorded as “0” before the intervention and “1” after the intervention, with July 2022 (i.e., time point = 37) as the intervention node; $X_{\text{posttime}}$ denotes the time count variable after the occurrence of the policy, with “0” denoting the pre-intervention period and “1, 2, 3……, 11” denoting the post-intervention period. $\varepsilon_{t}$ is the random error term.

$\beta_{0}$ denotes the pre-policy inpatient costs; $\beta_{1}$ represents the trend of the expenses before the policy was implemented; $\beta_{2}$ denotes the difference between the outcome and the counterfactual outcome when the policy is assumed to have been implemented, representing the immediate effect of the policy; and $\beta_{3}$ is the amount of change in slope, indicating the policy implementation’s impact on costs in the long term. All statistical analyses were performed using Stata, version 17. The level of significance was set at *p* < 0.05.

## 3 Results

### 3.1 Basic information of patients with osteoporotic hip fractures during 6 years

This study included 82,640 osteoporotic hip fracture patients in Shanghai: 30,944 men and 51,696 women, mostly over 65 years old. 36.3% of patients were hospitalized for 8–14 days, while merely 5.3% spent 31 days or more. Over half of the patients underwent surgery, most commonly third-degree (46.9%) and fourth-degree (33.6%). Most patients were admitted to grade-A tertiary hospitals (59.9%), then grade-B secondary hospitals (36.2%), with the fewest hospitalized in primary hospitals (3.9%). Besides, 59.9% of patients’ medical costs were covered by the Urban Employee Basic Medical Insurance (UEBMI), whereas only 13.8% were covered by the Urban–Rural Residents Basic Medical Insurance (URRBMI) (Table 1).

**Table 1:** Basic information of osteoporotic hip fracture patients in Shanghai within the years 2017–2022.

Characteristic	2017		2018		2019		2020		2021		2022		Total	
	Number	Percentage	Number	Percentage	Number	Percentage	Number	Percentage	Number	Percentage	Number	Percentage	Number	Percentage
Gender														
Male	5,331	38.9%	5,245	37.0%	5,565	37.8%	4,598	36.5%	5,408	37.5%	4,797	37.0%	30,944	37.4%
Female	8,378	61.1%	8,949	63.0%	9,170	62.2%	8,008	63.5%	9,017	62.5%	8,174	63.0%	51,696	62.6%
Age group														
≤44 years old	1,241	9.1%	1,172	8.3%	1,167	7.9%	917	7.3%	1,125	7.8%	1,003	7.7%	6,625	8.0%
45–64 years old	2,758	20.1%	2,659	18.7%	2,702	18.3%	2,231	17.7%	2,435	16.9%	2,047	15.8%	14,832	17.9%
≥65 years old	9,710	70.8%	10,363	73.0%	10,866	73.7%	9,458	75.0%	10,865	75.3%	9,921	76.5%	61,183	74.0%
Length of stay														
≤7 days	3,725	27.2%	3,718	26.2%	4,342	29.5%	3,784	30.0%	4,494	31.2%	4,407	34.0%	24,470	29.6%
8–14 days	4,717	34.4%	5,088	35.8%	5,252	35.6%	4,495	35.7%	5,537	38.4%	4,901	37.8%	29,990	36.3%
15–30 days	4,526	33.0%	4,552	32.1%	4,403	29.9%	3,675	29.2%	3,682	25.5%	2,946	22.7%	23,784	28.8%
≥31 days	741	5.4%	836	5.9%	738	5.0%	652	5.2%	712	4.9%	717	5.5%	4,396	5.3%
Surgery level														
Unoperated	2,226	16.2%	2,209	15.6%	2,135	14.5%	1,570	12.5%	1,631	11.3%	1,358	10.5%	11,129	13.5%
First-degree surgery	159	1.2%	185	1.3%	232	1.6%	233	1.8%	192	1.3%	195	1.5%	1,196	1.4%
Second-degree surgery	1,150	8.4%	800	5.6%	812	5.5%	443	3.5%	310	2.1%	254	2.0%	3,769	4.6%
Third-degree surgery	6,565	47.9%	7,211	50.8%	7,304	49.6%	5,609	44.5%	6,286	43.6%	5,806	44.8%	38,781	46.9%
Fourth-degree surgery	3,609	26.3%	3,789	26.7%	4,252	28.9%	4,751	37.7%	6,006	41.6%	5,358	41.3%	27,765	33.6%
Hospital level														
Primary hospital	417	3.0%	508	3.6%	516	3.5%	419	3.3%	603	4.2%	509	3.9%	2,972	3.6%
Grade-B secondary hospital	5,588	40.8%	5,742	40.5%	5,749	39.0%	5,134	40.7%	5,530	38.3%	4,692	36.2%	32,435	39.2%
Grade-A tertiary hospital	7,704	56.2%	7,944	56.0%	8,470	57.5%	7,053	55.9%	8,292	57.5%	7,770	59.9%	47,233	57.2%
Insurance														
Medical costs covered by the Urban Employee Basic Medical Insurance	7,835	57.2%	8,587	60.5%	8,855	60.1%	7,617	60.4%	8,631	59.8%	7,973	61.5%	49,498	59.9%
Medical costs covered by the Urban–Rural Residents Basic Medical Insurance	2,088	15.2%	1,840	13.0%	1,982	13.5%	1,817	14.4%	1,952	13.5%	1,785	13.8%	11,464	13.9%
Medical costs not covered by any health insurance	3,603	26.3%	3,449	24.3%	3,487	23.7%	2,753	21.8%	3,221	22.3%	2,552	19.7%	19,065	23.1%
Medical costs covered by other insurance	183	1.3%	318	2.2%	411	2.8%	419	3.3%	621	4.3%	661	5.1%	2,613	3.2%

### 3.2 Composition of hospitalization costs among patients with osteoporotic hip fractures

**Figure 1:**
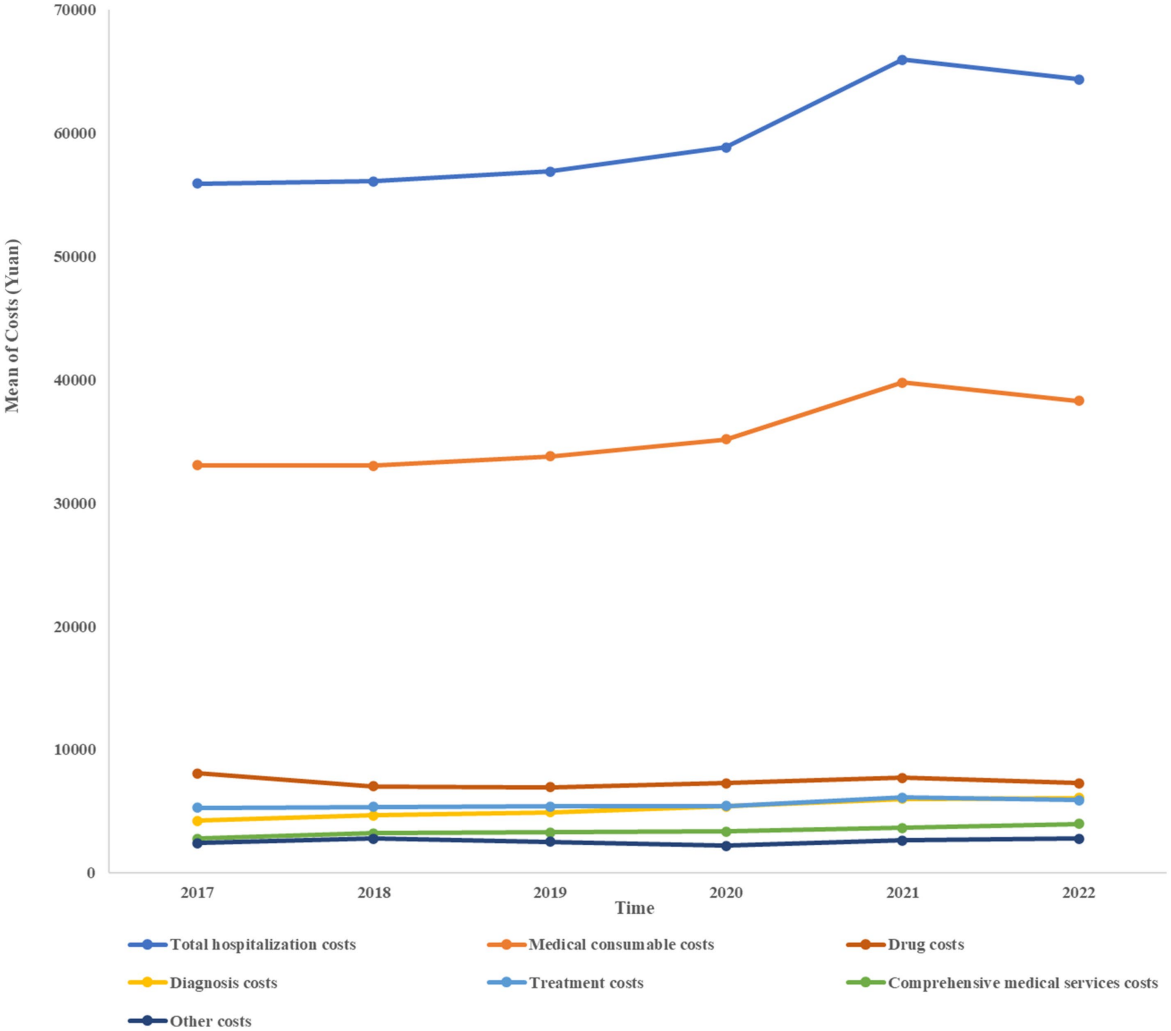
The time trend of the mean costs.

Medical consumable costs account for the most significant proportion of osteoporotic hip fracture inpatient costs each year, with the highest being 60.30% and 59.74% in 2021 and 2020, respectively, according to Table 2. Despite a declining tendency in 2022, medical consumable costs remained over 59% of the total expenditures. Between 2017 and 2022, the cost of drugs has shown an overall downward trend, from 14.48% in 2017 to 11.34% in 2022, ranking second. Table 2 and Figure 1 illustrate the composition of hospitalization expenses and their trends over the years, including expenditures of comprehensive medical services, diagnosis, treatment, drug, medical consumable, and other costs.

**Table 2:** Composition of hospitalization costs of patients with osteoporotic hip fractures in Shanghai within the years 2017–2022.

Year	Average hospitalization costs per admission (Yuan)	Comprehensive medical services costs		Diagnosis costs		Treatment costs		Drug costs		Medical consumable costs		Other costs	
		Costs (Yuan)	Percentage (%)	Costs (Yuan)	Percentage (%)	Costs (Yuan)	Percentage (%)	Costs (Yuan)	Percentage (%)	Costs (Yuan)	Percentage (%)	Costs (Yuan)	Percentage (%)
2017	55,923.39	2,800.23	5.01	4,231.87	7.57	5,293.21	9.47	8,099.84	14.48	33,069.59	59.13	2,428.66	4.34
2018	56,089.96	3,216.13	5.73	4,681.36	8.35	5,359.21	9.55	7,030.32	12.53	33,021.90	58.87	2,781.06	4.96
2019	56,893.18	3,306.94	5.81	4,934.68	8.67	5,394.08	9.48	6,952.49	12.22	33,783.59	59.38	2,521.39	4.43
2020	58,875.09	3,375.41	5.73	5,397.80	9.17	5,432.79	9.23	7,296.27	12.39	35,174.19	59.74	2,198.64	3.73
2021	65,953.77	3,649.20	5.53	6,019.07	9.13	6,139.93	9.31	7,723.90	11.71	39,772.39	60.30	2,649.29	4.02
2022	64,390.20	3,997.17	6.21	6,100.65	9.47	5,924.65	9.20	7,300.25	11.34	38,288.74	59.46	2,778.75	4.32

According to the hospital-level stratification results, the share of medical consumable costs was highest in grade-A tertiary hospitals, exceeding 60% in all 6 years. In contrast, primary hospitals had a share of medical consumable costs lower than 30%, while the share of drug costs was over 25%, except in 2022. The proportion of medical consumable costs in grade-B secondary hospitals ranged between grade-A tertiary and primary hospitals, at approximately 57%; meanwhile, drug costs were higher than in grade-A tertiary hospitals but lower than in primary hospitals (Tables 3–5).

**Table 3:** Composition of hospitalization costs of patients with osteoporotic hip fractures in Shanghai grade-A tertiary hospitals within the years 2017–2022.

Year	Average hospitalization costs per admission (Yuan)	Comprehensive medical services costs		Diagnosis costs		Treatment costs		Drug costs		Medical consumable costs		Other costs	
		Costs (Yuan)	Percentage (%)	Costs (Yuan)	Percentage (%)	Costs (Yuan)	Percentage (%)	Costs (Yuan)	Percentage (%)	Costs (Yuan)	Percentage (%)	Costs (Yuan)	Percentage (%)
2017	59,824.98	2,575.01	4.30	4,351.63	7.27	5,703.86	9.53	7,358.08	12.30	37,771.42	63.14	2,064.97	3.45
2018	59,961.05	2,849.84	4.75	4,777.07	7.97	5,849.24	9.76	6,509.18	10.86	37,516.44	62.57	2,459.27	4.10
2019	60,508.58	2,878.91	4.76	4,985.37	8.24	5,890.25	9.73	6,481.09	10.71	38,101.13	62.97	2,171.82	3.59
2020	62,594.51	2,968.79	4.74	5,496.26	8.78	5,953.36	9.51	6,843.43	10.93	39,419.53	62.98	1,913.14	3.06
2021	70,167.10	3,129.02	4.46	6,244.66	8.90	6,902.37	9.84	7,330.08	10.45	44,283.96	63.11	2,277.01	3.25
2022	67,172.50	3,146.89	4.68	6,097.19	9.08	6,427.10	9.57	6,817.52	10.15	42,053.62	62.61	2,630.19	3.92

**Table 4:** Composition of hospitalization costs of patients with osteoporotic hip fractures in Shanghai grade-B secondary hospitals within the years 2017–2022.

Year	Average hospitalization costs per admission (Yuan)	Comprehensive medical services costs		Diagnosis costs		Treatment costs		Drug costs		Medical consumable costs		Other costs	
		Costs (Yuan)	Percentage (%)	Costs (Yuan)	Percentage (%)	Costs (Yuan)	Percentage (%)	Costs (Yuan)	Percentage (%)	Costs (Yuan)	Percentage (%)	Costs (Yuan)	Percentage (%)
2017	51,847.39	2,902.21	5.60	4,070.93	7.85	4,881.69	9.42	8,959.25	17.28	28,470.02	54.91	2,563.28	4.94
2018	51,781.92	3,165.62	6.11	4,573.25	8.83	4,915.18	9.49	7,420.75	14.33	28,954.55	55.92	2,752.58	5.32
2019	52,441.26	3,381.27	6.45	4,895.97	9.34	4,810.21	9.17	7,211.79	13.75	29,501.70	56.26	2,640.31	5.03
2020	53,791.50	3,349.20	6.23	5,188.96	9.65	4,849.70	9.02	7,295.94	13.56	30,919.89	57.48	2,187.80	4.07
2021	60,867.40	3,517.33	5.78	5,621.89	9.24	5,343.35	8.78	7,623.49	12.52	36,302.91	59.64	2,458.44	4.04
2022	59,283.77	4,067.01	6.86	5,879.27	9.92	5,212.43	8.79	7,454.74	12.57	34,536.69	58.26	2,133.63	3.60

**Table 5:** Composition of hospitalization costs of patients with osteoporotic hip fractures in Shanghai primary hospitals within the years 2017–2022.

Year	Average hospitalization costs per admission (Yuan)	Comprehensive medical services costs		Diagnosis costs		Treatment costs		Drug costs		Medical consumable costs		Other costs	
		Costs (Yuan)	Percentage (%)	Costs (Yuan)	Percentage (%)	Costs (Yuan)	Percentage (%)	Costs (Yuan)	Percentage (%)	Costs (Yuan)	Percentage (%)	Costs (Yuan)	Percentage (%)
2017	38,462.66	5,594.46	14.55	4,175.88	10.86	3,220.98	8.37	10,287.30	26.75	7,840.45	20.38	7,343.59	19.09
2018	44,249.20	9,514.82	21.50	4,406.70	9.96	2,715.07	6.14	10,766.57	24.33	8,711.08	19.69	8,134.95	18.38
2019	47,148.29	9,504.69	20.16	4,533.97	9.62	3,754.77	7.96	11,801.39	25.03	10,619.01	22.52	6,934.46	14.71
2020	58,555.61	10,541.08	18.00	6,299.39	10.76	3,814.65	6.51	14,922.98	25.49	15,840.34	27.05	7,137.17	12.19
2021	54,661.36	12,011.65	21.97	6,559.21	12.00	2,960.75	5.42	14,060.19	25.72	9,550.73	17.47	9,518.82	17.41
2022	68,989.31	16,333.10	23.67	8,194.23	11.88	4,819.79	6.99	13,245.17	19.20	15,403.65	22.33	10,993.36	15.93

### 3.3 Structural variations of hospitalization costs among patients with osteoporotic hip fractures

The overall trend of drug costs as a proportion of total hospitalization costs has declined over the 6-year period for the VSV, except for 2019–2020. The drug costs had not only the highest contribution rate to overall inpatient expenses during the years 2017–2018 (44.22%) and 2020–2021 (36.76%), but it also ranked first in the CRSV from 2017 to 2022 (45.77%). Furthermore, although the share of medical consumable costs has been increasing for most years, it decreased in 2017–2018 and 2021–2022, with VSV of −0.26 and −0.84, respectively. While the reduction in medical consumable costs accounted for only 5.9% of the changes in the structure of inpatient costs during 2017–2018, their share showed the highest decline in the CRSV for 2021–2022 (31.82%) (Table 6).

**Table 6:** The value, degree, and contribution rate of structure variation of hospitalization costs within the years 2017–2022.

Year	Comprehensive medical services costs		Diagnosis costs		Treatment costs		Drug costs		Medical consumable costs		Other costs		DSV (%)
	VSV	CRSV (%)	VSV	CRSV (%)	VSV	CRSV (%)	VSV	CRSV (%)	VSV	CRSV (%)	VSV	CRSV (%)	
2017–2018	0.72	16.33	0.78	17.69	0.08	1.81	−1.95	44.22	−0.26	5.90	0.62	14.06	4.41
2018–2019	0.08	4.40	0.32	17.58	−0.07	3.85	−0.31	17.03	0.51	28.02	−0.53	29.12	1.82
2019–2020	−0.08	3.88	0.50	24.27	−0.25	12.14	0.17	8.25	0.36	17.48	−0.70	33.98	2.06
2020–2021	−0.20	10.81	−0.04	2.16	0.08	4.32	−0.68	36.76	0.56	30.27	0.29	15.68	1.85
2021–2022	0.68	25.76	0.34	12.88	−0.11	4.17	−0.37	14.02	−0.84	31.82	0.30	11.36	2.64
2017–2022	1.20	17.49	1.90	27.70	−0.27	3.94	−3.14	45.77	0.33	4.81	−0.02	0.29	6.86

The stratification results at the hospital level indicated that, while the share of drug expenses in tertiary and secondary hospitals exhibited an overall decreasing trend over the 6 years, primary hospitals reflected a decrease in only a few years. Additionally, medical consumable costs for both tertiary and secondary hospitals decreased in 2021–2022, with CRSVs of 23.36% and 37.91%, respectively; however, primary hospitals presented higher medical consumable costs during that period (Tables 7–9).

**Table 7:** The value, degree, and contribution rate of structure variation of hospitalization costs in Shanghai grade-A tertiary hospitals within the years 2017–2022.

Year	Comprehensive medical services costs		Diagnosis costs		Treatment costs		Drug costs		Medical consumable costs		Other costs		DSV (%)
	VSV	CRSV (%)	VSV	CRSV (%)	VSV	CRSV (%)	VSV	CRSV (%)	VSV	CRSV (%)	VSV	CRSV (%)	
2017–2018	0.45	11.14	0.70	17.33	0.23	5.69	−1.44	35.64	−0.57	14.11	0.65	16.09	4.04
2018–2019	0.01	0.73	0.27	19.71	−0.03	2.19	−0.15	10.95	0.40	29.20	−0.51	37.23	1.37
2019–2020	−0.02	1.30	0.54	35.06	−0.22	14.29	0.22	14.29	0.01	0.65	−0.53	34.42	1.54
2020–2021	−0.28	18.30	0.12	7.84	0.33	21.57	−0.48	31.37	0.13	8.50	0.19	12.42	1.53
2021–2022	0.22	10.28	0.18	8.41	−0.27	12.62	−0.30	14.02	−0.50	23.36	0.67	31.31	2.14
2017–2022	0.38	7.06	1.81	33.64	0.04	0.74	−2.15	39.96	−0.53	9.85	0.47	8.74	5.38

**Table 8:** The value, degree, and contribution rate of structure variation of hospitalization costs in Shanghai grade-B secondary hospitals within the years 2017–2022.

Year	Comprehensive medical services costs		Diagnosis costs		Treatment costs		Drug costs		Medical consumable costs		Other costs		DSV (%)
	VSV	CRSV (%)	VSV	CRSV (%)	VSV	CRSV (%)	VSV	CRSV (%)	VSV	CRSV (%)	VSV	CRSV (%)	
2017–2018	0.51	8.64	0.98	16.61	0.07	1.19	−2.95	50.00	1.01	17.12	0.38	6.44	5.90
2018–2019	0.34	14.29	0.51	21.43	−0.32	13.45	−0.58	24.37	0.34	14.29	−0.29	12.18	2.38
2019–2020	−0.22	7.21	0.31	10.16	−0.15	4.92	−0.19	6.23	1.22	40.00	−0.96	31.48	3.05
2020–2021	−0.45	10.39	−0.41	9.47	−0.24	5.54	−1.04	24.02	2.16	49.88	−0.03	0.69	4.33
2021–2022	1.08	29.67	0.68	18.68	0.01	0.27	0.05	1.37	−1.38	37.91	−0.44	12.09	3.64
2017–2022	1.26	9.43	2.07	15.49	−0.63	4.72	−4.71	35.25	3.35	25.07	−1.34	10.03	13.36

**Table 9:** The value, degree, and contribution rate of structure variation of hospitalization costs in Shanghai primary hospitals within the years 2017–2022.

Year	Comprehensive medical services costs		Diagnosis costs		Treatment costs		Drug costs		Medical consumable costs		Other costs		DSV (%)
	VSV	CRSV (%)	VSV	CRSV (%)	VSV	CRSV (%)	VSV	CRSV (%)	VSV	CRSV (%)	VSV	CRSV (%)	
2017–2018	6.95	50.00	−0.90	6.47	−2.23	16.04	−2.42	17.41	−0.69	4.96	−0.71	5.11	13.90
2018–2019	−1.34	12.52	−0.34	3.18	1.82	17.01	0.70	6.54	2.83	26.45	−3.67	34.30	10.70
2019–2020	−2.16	17.62	1.14	9.30	−1.45	11.83	0.46	3.75	4.53	36.95	−2.52	20.55	12.26
2020–2021	3.97	18.61	1.24	5.81	−1.09	5.11	0.23	1.08	−9.58	44.91	5.22	24.47	21.33
2021–2022	1.70	10.46	−0.12	0.74	1.57	9.66	−6.52	40.12	4.86	29.91	−1.48	9.11	16.25
2017–2022	9.12	37.72	1.02	4.22	−1.38	5.71	−7.55	31.22	1.95	8.06	−3.16	13.07	24.18

According to DSV, 2017–2018 marked the year with the most significant structural change in individual hospitalization costs, which applied to overall costs and the stratification of tertiary and secondary hospital costs (Tables 6–8). During this period, drug and medical consumable costs decreased, while other expenses, such as diagnosis costs, remained elevated. However, for secondary hospitals, the percentage of medical consumable costs showed an elevated trend in 2017–2018, despite a decline in drug costs. The 2021–2022 and 2019–2020 presented the second and third highest DSV for overall costs, respectively (Table 6).

### 3.4 New gray relational analysis of correlation between total and individual hospitalization costs

From 2017 to 2022, medical consumable costs consistently showed the highest correlation with total hospitalization costs, averaging a correlation of 0.977, which is nearly 1. The degree of correlation between drug costs and total hospitalization costs has declined over time, but it remained second in the correlation rankings. Treatment costs, on the other hand, were the third most correlated, only slightly above diagnosis costs (Table 10).

**Table 10:** The coefficient, degree and order of correlation between total and individual hospitalization costs within the years 2017–2022.

Year	Correlation coefficient					
	Comprehensive medical services costs	Diagnosis costs	Treatment costs	Drug costs	Medical consumable costs	Other costs
2017	0.64	0.65	0.66	0.69	1.00	0.64
2018	0.64	0.66	0.66	0.68	1.00	0.64
2019	0.64	0.65	0.66	0.67	1.00	0.63
2020	0.63	0.64	0.64	0.65	0.98	0.62
2021	0.58	0.60	0.60	0.61	0.94	0.57
2022	0.59	0.61	0.60	0.61	0.94	0.58
Correlation degree	0.620	0.635	0.637	0.652	0.977	0.613
Correlation order	5	4	3	2	1	6

Based on the subgroup analysis at the hospital level, medical consumable costs topped the correlation order for tertiary and secondary hospitals, which was in line with the overall analysis; however, diagnosis costs replaced treatment costs as third among secondary hospitals. Notably, the drug costs, rather than medical consumable costs, had the highest correlation degree with total hospitalization costs in primary hospitals; meanwhile, the ranking of comprehensive medical services costs rose, while the ranking of diagnosis and treatment costs decreased (Tables 11–13).

**Table 11:** The coefficient, degree and order of correlation between total and individual hospitalization costs in Shanghai grade-A tertiary hospitals within the years 2017–2022.

Year	Correlation coefficient					
	Comprehensive medical services costs	Diagnosis costs	Treatment costs	Drug costs	Medical consumable costs	Other costs
2017	0.61	0.63	0.64	0.65	1.00	0.61
2018	0.61	0.63	0.64	0.64	0.99	0.61
2019	0.61	0.63	0.63	0.64	0.99	0.61
2020	0.6	0.62	0.62	0.62	0.98	0.59
2021	0.55	0.57	0.58	0.58	0.94	0.55
2022	0.57	0.59	0.59	0.59	0.95	0.57
Correlation degree	0.592	0.612	0.617	0.620	0.975	0.590
Correlation order	5	4	3	2	1	6

**Table 12:** The coefficient, degree and order of correlation between total and individual hospitalization costs in Shanghai grade-B secondary hospitals within the years 2017–2022.

Year	Correlation coefficient					
	Comprehensive medical services costs	Diagnosis costs	Treatment costs	Drug costs	Medical consumable costs	Other costs
2017	0.67	0.68	0.68	0.72	0.99	0.66
2018	0.67	0.68	0.68	0.71	1.00	0.67
2019	0.66	0.68	0.68	0.70	1.00	0.66
2020	0.65	0.67	0.67	0.69	1.00	0.64
2021	0.60	0.62	0.61	0.63	0.97	0.59
2022	0.62	0.63	0.62	0.64	0.96	0.60
Correlation degree	0.645	0.660	0.657	0.682	0.987	0.637
Correlation order	5	3	4	2	1	6

**Table 13:** The coefficient, degree and order of correlation between total and individual hospitalization costs in Shanghai primary hospitals within the years 2017–2022.

Year	Correlation coefficient					
	Comprehensive medical services costs	Diagnosis costs	Treatment costs	Drug costs	Medical consumable costs	Other costs
2017	0.93	0.91	0.90	1.00	0.96	0.95
2018	0.90	0.84	0.82	0.92	0.89	0.88
2019	0.86	0.81	0.80	0.89	0.88	0.83
2020	0.75	0.71	0.69	0.80	0.81	0.72
2021	0.81	0.75	0.72	0.83	0.78	0.78
2022	0.71	0.65	0.63	0.69	0.70	0.67
Correlation degree	0.827	0.778	0.760	0.855	0.837	0.805
Correlation order	3	5	6	1	2	4

### 3.5 The impact of the national centralized volume-based procurement policy on overall costs and their structure

**Figure 2:**
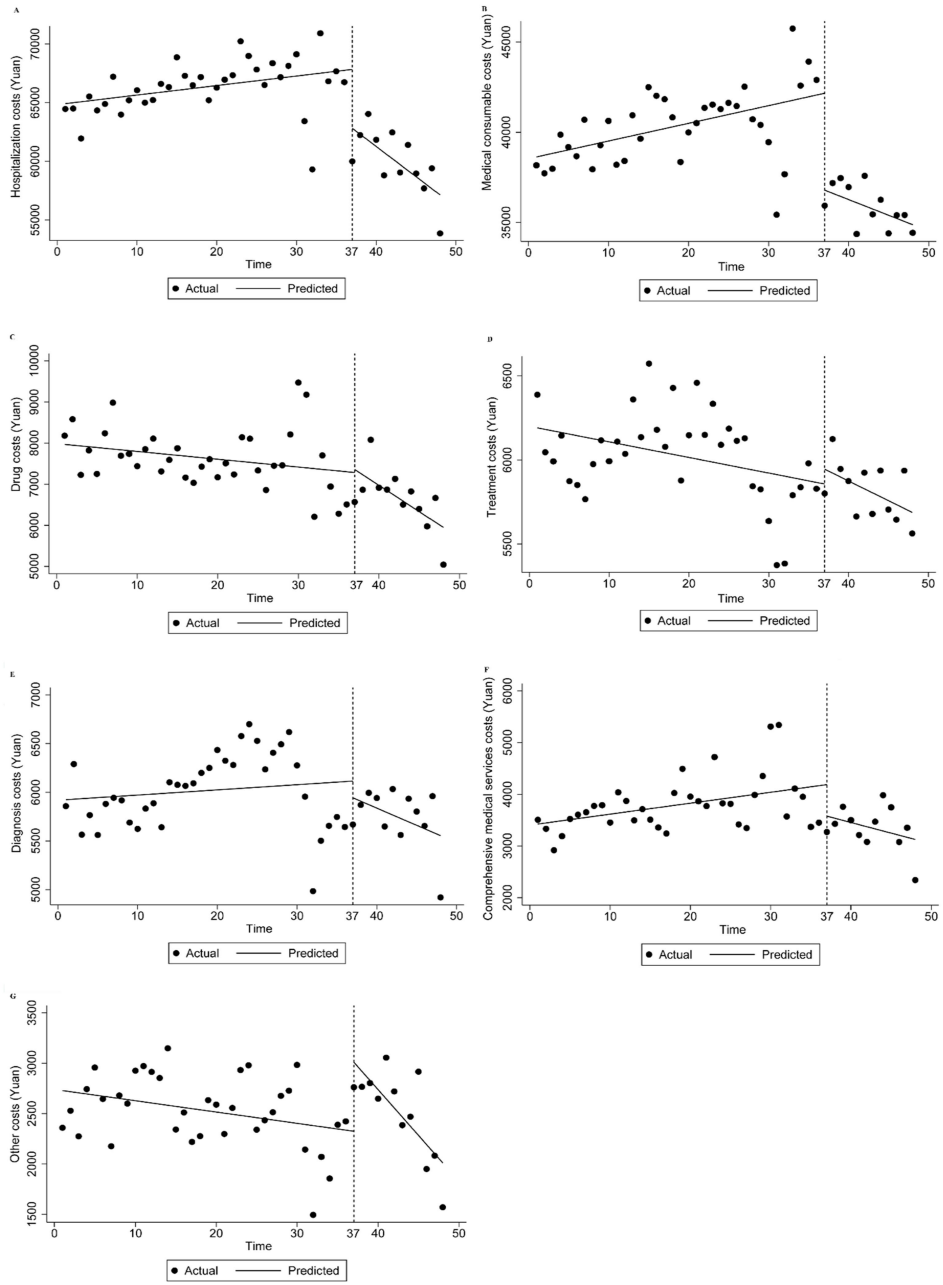
Trend in mean of total costs and its structure of inpatients of osteoporotic hip fractures from 2021 to 2022. Median of expense of inpatient **(A)** total hospitalization costs, **(B)** medical consumable costs, **(C)** drug costs, **(D)** treatment costs, **(E)** diagnosis costs, **(F)** comprehensive medical services costs, **(G)** other costs.

The effects of the policy on total hospitalization costs and their various components were shown in Table 14 and Figure 2. Before the reform, the mean total hospitalization and medical consumable costs increased by 81.075 and 94.095 yuan (*p* < 0.05), respectively. After the NVBP policy was implemented, total hospitalization costs significantly decreased by 5,022.088 yuan (*p* < 0.001). Additionally, compared to the trend before the intervention, total hospitalization costs exhibited a significant downward trend over time, decreasing by 596.114 yuan after the intervention (*p* < 0.001). Similarly, medical consumable costs also exhibited a declining pattern regarding both immediate effects and long-term patterns after the reform, with an average decrease of 290.448 yuan (*p* < 0.001).

**Table 14:** The effect of the national centralized volume-based procurement policy on total costs and its structure.

Items of expense	Intercept (β_0_)		Slope pre-reform (β_1_)		Change in level due to reform (β_2_)		Change in slope due to reform (β_3_)	
	Coefficient	*p* value	Coefficient	*p* value	Coefficient	*p* value	Coefficient	*p* value
Total hospitalization costs	64,919.633	<0.001	81.075	0.028	−5,022.088	<0.001	−596.114	<0.001
Medical consumable costs	38,684.267	<0.001	94.095	0.020	−5,087.104	<0.001	−290.448	<0.001
Drug costs	7,967.544	<0.001	−19.018	0.216	73.336	0.867	−109.035	0.040
Treatment costs	6,190.860	<0.001	−9.276	0.102	88.044	0.541	−14.124	0.126
Diagnosis costs	5,924.064	<0.001	5.292	0.595	−171.088	0.523	−40.640	0.131
Comprehensive medical services costs	3,424.492	<0.001	21.189	0.057	−610.302	0.039	−62.265	0.109
Other costs	2,728.405	<0.001	−11.207	0.134	685.027	0.004	−79.602	0.006

Moreover, drug costs declined in the long run (*p* < 0.05), even though the policy’s immediate impact led to a cost-increase that was not statistically significant. Other costs rose during the initial stage after the reform was implemented (*p* < 0.05). Nevertheless, compared to before the intervention, these costs decreased by 79.602 yuan every half month in the long-term trend following the intervention (*p* < 0.05). Comprehensive medical services costs decreased after the policy was implemented (*p* < 0.05), but this decrease was not statistically significant in the long-term trend (Table 14; Figure 2).

## 4 Discussion

Based on a large sample size from 2017 to 2022, this study investigated the variations in hospitalization costs for patients with osteoporotic hip fractures in Shanghai over time and the extent to which individual costs relate to total expenses. Additionally, we stratified costs for patients hospitalized at various levels of hospitals to explore the variations in expenses and the correlation between total and individual hospitalization costs. Furthermore, this study assessed the instant and long-term impact of NVBP on hospitalization costs using interrupted time series. Overall, our research found that total hospitalization costs for osteoporotic hip fracture patients displayed an increasing trend, while drug costs decreased during this period, along with a significant drop in medical consumable costs between 2021 and 2022. Despite the NVBP’s effectiveness, medical consumable costs remain a major source of costly expenditures for inpatients with hip fractures. The structural variation in hospitalization costs differed by the level of medical institutions, possibly due to the policy’s varying impact between primary and higher-grade hospitals.

Our study demonstrated that the medical consumable costs dominated hospitalization expenses for patients with osteoporotic hip fractures. This may be due to the fact that fracture treatment often requires expensive surgical consumables (e.g., artificial joints, dynamic hip screws, or proximal femoral nail anti-rotation instruments), which is a concern not only in the treatment of fractures but also in their management (15, 35). Nevertheless, the analysis of structural variations of costs showed a significant reduction in the medical consumable costs in 2021–2022, which had the highest contribution rate to the variation of the expenses in that year. This may result from a series of policies on controlling the price of medical consumables that are gradually taking effect (28, 30–32). Further analysis of the impact of the NVBP policy on the medical consumable costs revealed that the reform has led to a decrease in the medical consumable costs, both in terms of the immediate effects of the reform and the long-term trend. Meanwhile, the policy has significantly reduced total hospitalization costs, suggesting that it has achieved the desired effect. Li et al. explored the impact of the NVBP in Guangdong Province on inpatient costs for total knee arthroplasty patients and found that overall expenses and medical consumables costs decreased after the policy, which supports the policy’s effects from another perspective (39). However, the gray correlation analysis revealed that in 2022, medical consumable costs were still the highest among individual hospitalization costs, indicating that controlling these costs played a key role in alleviating the impact of disease on patients suffering from osteoporotic hip fractures. High medical consumable costs can restrict access to essential treatments for patients with osteoporotic hip fractures, especially for those in rural China, impacting patient health outcomes (35). A cross-sectional study conducted in rural China revealed evident socioeconomic disparities, with insured individuals and homeowners more likely to receive assistance after fractures (36). The study also indicated that medical interventions, including supplement use and osteoporosis treatment, were crucial to recovery and were significantly associated with higher chances of receiving help after osteoporotic fractures (36, 40).

Drug costs have shown a general trend of reduction over the six-year period, with their reduction being the primary contributor to the overall cost change, especially during 2017–2018. In Japan, total medical costs for hip fracture patients gradually rose after an initial decline from 2011 to 2021, while drug costs decreased—a trend similar to what this study found in China (41). Hospitalization costs for osteoporotic hip fractures in Thailand also increased by 2.5 times between 2013 and 2022, whereas drug costs declined (42). To control drug costs, the central government of China has implemented a range of measures, including a zero-markup drug policy, which has gradually reduced drug revenues for public hospitals since its implementation (19). After September 2017, the policy allowing markups on drug prices was completely abolished for all public hospitals, which may explain the significant decrease in drug costs from 2017 to 2018 in this study. This study’s results were consistent with other studies conducted in China, indicating a reduction in drug costs in the years following the policy’s implementation (26, 27, 29). Wang et al. found that, compared to a fixed percent mark-up drug policy, a transitional policy that allows drugs to be sold at a fixed mark-up percent, zero-markup drug policy was more intensive, especially regarding the decline of outpatient drug costs (27). However, research indicated that hospitals have pursued other income sources to compensate for the decline in drug revenue (43, 44). This study also observed a similar phenomenon: although drug costs have decreased, comprehensive service fees and diagnostic fees have increased, indicating a shift in costs. Meanwhile, total hospitalization costs have risen yearly, except for a decline in 2022, which aligns with a study conducted in Tangshan, China (36). This suggests that further policies are needed to improve earlier strategies.

Previous study has noted that the type of hospital is associated with total cost expenditures for osteoporotic hip fractures (20). The subgroup analysis results of this study showed that the structure of hospitalization costs for patients with osteoporotic hip fractures varied across different levels of hospital. In addition, compared to tertiary medical institutions, patients in primary medical institutions had a higher proportion of drug costs and a lower proportion of medical consumable costs. Meanwhile, the results of the gray correlation analysis suggest that, in contrast to other levels of medical institutions, the correlation between comprehensive medical services costs and total hospitalization costs is higher in first-level hospitals. Due to the varying functional roles of healthcare institutions in China, primary total hospitals mainly provide basic medical services and chronic disease management, tertiary hospitals primarily treat complex and severe cases, while secondary hospitals handle patients referred from tertiary hospitals (45). As a result, patients with osteoporotic hip fractures often undergo surgical treatment at higher level hospitals but choose to have rehabilitation at primary hospitals, leading to differences in the structure of inpatient costs.

The changes in the cost structure of medical institutions at various levels also differ. While the medical consumable costs at secondary and tertiary hospitals showed a notable decrease in 2021–2022, the proportion of medical consumable costs at primary hospitals increased. This may be because the NVBP policy implemented in 2022 mainly targets high-value consumables used in joint replacement surgery, and does not involve medical consumables required for orthopedic rehabilitation, thus having little impact on the medical consumable costs for osteoporotic hip fractures in primary hospitals (30). Beyond medical consumable costs, our results also revealed that although drug costs in tertiary and secondary hospitals have generally decreased over 6 years, primary hospitals have only shown a decline in a few years. This can be explained by higher-level hospitals receiving relatively more financial subsidies, along with a more comprehensive compensation mechanism from the government (19). As a result, after implementing the zero-markup drug policy, these institutions managed to counter the decline in drug revenue more swiftly. They achieved this by adjusting revenue composition and receiving government subsidies, which significantly lowered the share of drug costs. In addition, stricter government regulations for higher-level hospitals compared to lower-level hospitals, along with more sophisticated management practices and advanced information systems in higher-level hospitals, can also clarify the effectiveness of policy implementation (46). Furthermore, this study discovered that the proportion of drug costs in primary hospitals significantly decreased in 2021–2022, suggesting some degree of policy lag. In the context of China’s hierarchical diagnosis and treatment reforms, higher-level hospitals have become the de facto centers of excellence for complex conditions like osteoporotic hip fractures, concentrating resources such as high-volume surgical experience, state-of-the-art operating theaters, subspecialty teams, etc. This centralization can improve the quality of care for patients with severe conditions who choose to seek treatment in these hospitals. However, it may also lead to a situation where primary hospitals, which primarily focus on rehabilitation and less complex cases, face higher relative costs for medical consumables and drugs. This could limit the access of patients in primary hospitals to necessary treatments, particularly those requiring high-value consumables. Therefore, targeted policies should be developed to address the specific needs of primary hospitals, ensuring that they have the necessary resources to provide effective rehabilitation and follow-up care for patients with osteoporotic hip fractures.

The COVID-19 pandemic, which occurred during the study period, may have influenced healthcare cost patterns. However, a review of the literature shows that several studies with similar methodologies to ours have also not specifically addressed the impact of the COVID-19 pandemic (19, 37, 39). This indicates that although the pandemic may have had some impact, it is not regarded as a significant confounding factor in studies with similar methodologies. Future research should consider the pandemic’s influence and employ suitable statistical techniques to adjust for its effects when examining healthcare costs and policy changes.

Our study has several limitations. First, only direct medical costs were included in this study, excluding direct non-medical costs such as transportation and caregiving, which suggests that future research could take a more comprehensive look at the costs for people with osteoporotic hip fractures from a whole-society perspective (34, 47). Second, the analysis covered data only through 2022, making it challenging to analyze the long-term impact of multiple policies (48). However, the findings of this study still explore changes in hospitalization costs over an extended period, which could serve as a foundation for future research. Third, reliance on data obtained from the hospital reporting system may introduce information bias despite strict cleaning protocols. Therefore, it will be essential to utilize additional data sources in the future to validate this study’s findings further. Fourth, our study period includes the COVID-19 pandemic, which could have affected healthcare costs and hospitalization patterns. While we recognize this potential influence, we did not have detailed data on the specific effects of the pandemic. Future research should consider the pandemic’s impact and apply appropriate statistical methods to account for its effects. Fifth, comorbidities, fracture types, and surgical procedures could not be properly identified due to limitations in data gathering for this investigation. This may have had an impact on the cost analysis’s findings. To account for confounding variables and increase the precision of the results, future studies might conduct more subgroup analyses based on different comorbidities, fracture types, or surgical procedures.

## 5 Conclusion

Overall hospitalization costs for osteoporotic hip fracture patients increased from 2017 to 2022. Medical consumable costs continued to dominate the total expenses, although they showed a decrease due to the implementation of the NVBP. Despite the downward trend in drug costs, they remain the second-largest component of total hospitalization expenses. The stratification results based on the level of the healthcare institution indicate that the variation of the inpatient cost structure in primary hospitals differs from that of higher-grade hospitals. For the health system, addressing cost drivers, especially medical consumable costs and drug costs, is crucial for ensuring the sustainable use of healthcare resources and improving operational efficiency. For policymakers, corresponding measures should be taken in accordance with the cost structure characteristics of medical institutions at different levels to ensure equitable and effective cost management.

## Data Availability

The datasets presented in this article are not readily available because this dataset contain sensitive information that cannot be disclosed publicly. Requests to access the datasets should be directed to Jing Yan, yanjing@shdrc.org.
